# Vietnam military service history and prostate cancer

**DOI:** 10.1186/1471-2458-6-75

**Published:** 2006-03-24

**Authors:** Leavy Justine, Ambrosini Gina, Fritschi Lin

**Affiliations:** 1 School of Population Health, University of Western Australia, Crawley Western Australia 6009, Australia

## Abstract

**Background:**

Three decades after US and Australian forces withdrew from Vietnam, there has been much public interest in the health consequences of service in Vietnam. One controversial question is whether the risk of prostate cancer amongst Vietnam veterans is increased. This paper examines relationships between military history, family history and risk of prostate cancer in a population-based case control study.

**Methods:**

Cases were selected from the Cancer Registry of Western Australia as incident cases of histologically-confirmed prostate cancer, and controls were age-matched and selected from the Western Australian electoral roll. Study participants were asked to report any military service history and details about that service.

**Results:**

Between January 2001 and September 2002, 606 cases and 471 controls aged between 40–75 years were recruited. An increased prostate cancer risk was observed in men reporting they were deployed in Vietnam although this was not statistically significant (OR = 2.12; 95% CI 0.88–5.06). An increased risk was also observed in men reporting prostate cancer in fathers (OR = 1.90; 95% CI 1.20–3.00) or brothers (OR = 2.05; 95% CI 1.20–3.50) diagnosed with prostate cancer.

**Conclusion:**

These findings support a positive association between prostate cancer and military service history in the Vietnam war and a first degree relative family history of prostate cancer.

## Background

For more than two decades the perceived ill-health of Vietnam veterans has been a public issue in Australia. As part of the Allied Forces in Vietnam, over 50 000 Australians served from 1964–1972, with approximately 500 losing their lives and nearly 2500 wounded [1]. Vietnam veterans have been studied in Australia and elsewhere, in an attempt to explain how service-related experience may affect their health and well-being in both the short and long-term [2].

In 1998 the Australian Institute of Health and Welfare [2] surveyed male Vietnam veterans regarding their current health problems [3]. Medical confirmation of some of these self-reported medical conditions was attempted in 1999 [2]. A higher prevalence of several conditions (including prostate cancer) was found in Vietnam veterans compared to the Australian community standard [2].

In this study, we aimed to use a case-control design to examine whether service in the Vietnam war may be associated with an increased prostate cancer risk after adjusting for known risk factors such as age and family history.

## Methods

We used data from a population based case-control study undertaken in Perth, Western Australia during 2001–2002.

Cases were men between the ages of 40 and 75 years with prostate cancer, histologically confirmed between January 1, 2001 and August 30, 2002. Cases were identified from the Cancer Registry of Western Australia. Cancer reporting by pathologists is mandatory in Western Australia.

Permission was sought from each case's urologist before approaching the patient to participate in the study. During the study period we identified 1226 apparently-eligible prostate cancer cases and excluded 166. Reasons for exclusion included; case did not have a Western Australian contact address; admitting doctor and/or operating doctor and/or separation doctor could not be identified; or urologist did not reply. Of the 1066 men invited to be in the study 685 (64%) of cases gave their consent to be in the study. Cases for which the doctor refused permission were slightly more likely to be urban residents but had a similar age distribution to the cases for which we had permission to approach. Of those approached, there were no differences in age and rural/urban residence between participants and non-participants.

Controls were randomly selected from men aged 40–75 years, without a history of prostate cancer, and who were currently enrolled on the Electoral Roll of Western Australia. The controls were frequency matched to the expected case distribution within 5-year age groups. Of the 1272 controls invited to participate in the study, 547 (43%) gave their consent. We had no data on non-participating controls.

All study participants gave their informed consent before participating. The study was approved by the Ethics Committee of the University of Western Australia and the Confidentiality of Health Information Committee, Department of Health, Western Australia.

Eligible subjects received a reminder card if they had not returned their consent form within 21 days.

Consenting subjects were required to complete a self-administered questionnaire on personal demographic factors; family cancer history; screening history and occupational history. Subjects were asked to report if they had ever been a member of the military or national defence service as a regular or reserve member, and if yes, if they were ever deployed in an area of conflict. If they had been deployed in an area of conflict they were also asked to complete the following details: year deployed; duration of deployment; country or region of deployment; job title; rank; unit/ship/squadron name.

The Gleason's score assigned at the time of prostate cancer diagnosis was collected for each case from the pathology reports held by the Cancer Registry of Western Australia. The Gleason's score is a grading dependent on the histological architecture of the prostate cancer cell, and has been reported as the most valuable prognostic factor for prostate cancer cases [4]. The highest score possible is 10, indicating more aggressive disease and possibly a poor prognosis. 'Aggressive prostate cancer' was defined by a Gleason's score of greater than or equal to 7.

Data were analysed using contingency tables and chi-squared tests. Logistic regression was performed to calculate odds ratios (OR) and 95% confidence intervals (CI) for the risk of prostate cancer by family history and military service after adjusting for the effect of age. Data were analysed by statistical software package SPSS11 for Windows. We repeated the analyses restricting the prostate cancer cases to those with a Gleason's score of greater than or equal to 7.

## Results

Cases and controls were reasonably well matched by age (Table 1). Cases were more likely to report fathers or brothers with prostate cancer (Table 1). Family history had a significant positive association with prostate cancer. Having a father or brother with prostate cancer almost doubled the risk of prostate cancer (Table 2). Similarly, family history had an increased effect in the aggressive prostate cancer group, for a father with a history of prostate cancer the OR was 2.09; (95% CI 0.87 – 5.00) and for a brother with prostate cancer the OR was 2.13; (95% CI 0.89 – 5.10).

A history of military service, or service in an area of conflict did not increase the risk of prostate cancer (Table 1 and Table 2). However, having served in Vietnam almost doubled the risk of prostate cancer, although this was not statistically significant. After adjusting for family history, the risk of prostate cancer in those serving in Vietnam was 2.08; (95% CI 0.87 – 5.01). When the analysis was restricted to those with aggressive prostate cancer the results were similar with an OR of 2.09; (95% CI 0.79 – 5.49).

Of the Vietnam veterans in our study, 76.5% were Australian Regular Army (ARA) personnel; 17.6% Royal Australian Navy (RAN) personnel and 5.9% in the Royal Australian Air Force (RAAF). The most common serving rank was Private 29.4%, followed by Corporal 23.5% and Sergeant 17.6%. The other ranks were comprised of lance-corporal, leading aircraftsmen, warrant officer and captain. The average age of Vietnam veterans was 58.0 years, the average of non-Vietnam veterans was 65.0 years. The age range of veterans was between 50–74 years.

With regard to experiences after Vietnam there were no differences between Vietnam veterans who did or did not develop cancer with regard to country of birth, smoking status, body mass index, alcohol intake, years spent in farming or driving vehicles, or occupational physical activity.

## Discussion

This population based case-control study has confirmed that a family history among first degree relatives is an important risk factor for prostate cancer, and suggests that service in the Vietnam war is also a risk factor for prostate cancer (although this finding did not reach statistical significance).

The role of family history is one of the few factors that do have a consistent positive association with prostate cancer [5]. Almost universally studies using different study designs including hospital based and population based case-control, cross sectional, family and cohort studies have found that a family history of prostate cancer in a first degree relative is associated with at least a doubling of risk among relatives [6-11] similar to our finding.

Several limitations of our data on family history are acknowledged. Firstly, family history data was collected by self reports which were not verified. However self report of prostate cancer in first degree relatives has been shown to be relatively accurate [5,6]. Selection bias may also have occurred if controls with a family history of prostate cancer were more likely to participate in the case-control study. Recall bias may also have occurred, as cases may differentially report their family history compared with healthy controls.

In addition, the level of screening in the Australian population is an area of interest to note. In the early 1990s the incidence of prostate cancer dramatically increased after the introduction of widespread use of prostate-specific antigen (PSA) testing became fashionable in Australia [12-14]. These increases may have had an affect on the reporting of family history of prostate cancer [14].

Our analysis suggests a doubling of prostate cancer risk among Vietnam War veterans. However this result was not statistically significant and was based on small numbers of Vietnam veterans (n = 34). These results are consistent with the findings of the Australian veterans study [2] which validated veterans self-report of prostate cancer with the national cancer registry. It found 212 confirmed cases of prostate cancer as compared with 147 expected cases [2], which equates to a standardized incidence ratio of 144. One proposed causative factor for the increase in prostate cancer is exposure to herbicides from a US operation known as 'Ranch Hand'. Nearly 19 million gallons of herbicide were sprayed on approximately 3.6 million acres of Vietnamese land [15]. Spraying began in 1962, intensified in 1967 and was believed to be stopped in 1971 [16]. During the operation a variety of herbicide formulations were used, however most were mixtures of phenoxy herbicides, 2,4-dichlorophenoxyacteic acid (2,4-d) and 2,4,5-trichlorophenoxyacetic acid (2,4,5-T) [15,17]. This herbicide was shipped out in drums with orange stripes, and so it was called Agent Orange [15].

The Center for Disease Control (CDC) proposed three efforts to assess the health of Vietnam veterans in the 1980's including a historical cohort study that compared 9324 Vietnam Army veterans with 8989 Vietnam-era Army veterans that served elsewhere [18] and a related population based case-control study using incident cases from eight cancer registries [15]. However, the overall numbers of subjects in these studies with substantial herbicide exposure were too small to support firm conclusions [15,18,19].

In a cohort of Australian national service conscripts, cause of death classes for deaths among 19 205 veterans of the Vietnam conflict were compared to 25 677 veterans who only served in Australia. Of 260 deaths, thirty three were due to neoplasms [20]. There was no statistically significant difference between the two groups in death rates from neoplasms, nor were deaths from specific neoplasms more frequent among the group that served in Vietnam [20]. However this study was limited by the relatively brief follow-up period and the young age of the Vietnam veteran population and a follow-up study at a more relevant time was recommended [15,17,20]. Most of our subjects were in over 55 and thus are in the age-groups of most interest for any cancers that may have been induced by exposure in Vietnam [21].

One of the challenges in assessing the health outcomes of the Vietnam conflict is quantifying the exposure. There is little precise information about how much exposure or even what herbicides any individual was exposed to and no standardised methods are available for estimating the extent of the exposure on an individual level [15,22,23]. Other combat exposures such as infectious diseases and stress are also difficult to measure [22].

Of the veterans in our study that served in Vietnam, most were army personnel. The literature suggests that members of the US Army Chemical Corps, who stored and mixed herbicides are thought to have had the heaviest exposures; members of the Special Forces units who defoliated remote campsites; and members of Navy river units may also have had heavy exposures [15,22]. Australian troops tended to be confined to the Phoc Tuy Province which was not heavily sprayed [24].

Because of the limitations of the Vietnam veteran studies, indirect sources provide an important secondary source on the potential carcinogenicity of Agent Orange exposure. These include studies on Vietnamese soldiers exposed to the same herbicides; occupationally exposed workers in a variety of settings; people exposed after industrial accidents. On review of this literature, Frumkin (2003) suggested the evidence of an association between Agent Orange and prostate cancer is not strong.

In Australia the Department of Veterans Affairs (DVA) is responsible for providing health care to Australian veterans, including veterans who served in Vietnam [25]. Prior to the mid 1990's Australian Vietnam veterans received most of their health care in hospitals that were government owned and operated [26]. The veterans' health scheme now funds, rather than provides directly treatment by general practitioners, specialists, hospitals and allied health providers [25]. Some co-payments are required for high end dental and optical treatment [25]. Essentially veterans have access to health services that represent the best mix for an individuals' circumstance. Veteran status should not represent any distinct access or financial advantage over the general population in Australia who have access to a health care system which is a public-private mix.[25]

Strengths of our study include the use of a population-based cancer registry and pathologically-confirmed cases of prostate cancer. Further, the military history data we collected were detailed and not the sole focus of the self-administered questionnaire. The accuracy of self reported combat exposure and deployment in an area of conflict has been reported to be consistent amongst men who have held a tactical military occupational specialty, amongst those assigned to combat units and amongst those who served in Vietnam [22].

A limitation of this study was the low response from eligible participants. Only 64% of the eligible cases and 43% of eligible controls agreed to participate in this study. However, this response rate for controls is consistent with other recently conducted studies of Australian men and prostate cancer [12,27,28]. There did not appear to be major biases in the selection of cases, but we were unable to examine selection bias in the selection of controls.

## Conclusion

A family history of prostate cancer is an important risk factor amongst first degree relatives, and this confirms earlier studies [6-11].

The risk of prostate cancer appears to be increased among men who were deployed as military personnel in the Vietnam War after adjusting for age and family history. Given the number of men deployed, this has possible ramifications in terms of the number of prostate cases still to occur, compensation for illness, and burden on the health care system.

## Competing interests

The author(s) declare that they have no competing interests.

## Authors' contributions

All authors have made a contribution to the information or material submittted for publication. LF and GA are Chief Investigators for the Prostate Health Study, Western Australia. JL participated in the design of the study, case recruitment, data collection, Cancer Registry follow-up and drafted the manuscript. JL & LF performed statistical analysis and interpretation of the data. All authors have read and approved the final manuscript.

## Pre-publication history

The pre-publication history for this paper can be accessed here:



## Figures and Tables

**Table 1:** Characteristics of prostate cancer cases and controls

	**Cases n = 560 N(%)**	**Controls n = 450 N(%)**	**p-value**
**Age group (years)**			
50 and under	16 (2.9)	11 (2.4)	p < 0.00
51–55	67 (12)	17 (3.8)	
56–60	106 (18.9)	61 (13.6)	
61–65	126 (22.5)	122 (27.1)	
66–69	139 (24.8)	132 (29.3)	
70 and above	106 (18.9)	107 (23.8)	
**Father with prostate cancer**			
Yes	70 (12.5)	29 (6.4)	0.00
No	490 (87.5)	421 (93.6)	
**Brother with prostate cancer**			
Has brother No Cap	358 (63.9)	316 (70.2)	0.03
Has brother, Has CaP	47 (8.4)	22 (4.9)	
No brother	155 (27.7)	112 (24.9)	
**Served in military**			
Yes	227 (40.5)	179 (39.8)	0.80
No	333 (59.5)	271 (60.2)	
**Area Served**			
Vietnam			
Yes	25 (4.5)	7 (1.6)	0.00
No	535 (95.5)	443 (98.4)	
Korea			
Yes	4 (.7)	8 (1.6)	0.12
No	556 (99.3)	442 (98.2)	
South East (SE) Asia			
Yes	43 (7.7)	31 (6.9)	0.63
No	517 (92.3)	419 (93.1)	

**Table 2:** Odds ratios (OR) and 95% confidence intervals (CI) for risk of prostate cancer in relation to family history and military combat history (all adjusting for age).

	OR*	**95% CI**	**p-value**
**Family History**			
Father with prostate cancer	1.90	1.20–3.00	0.007
Brother with prostate cancer	2.05	1.20–3.50	0.008

**Served in military **	1.14	0.88 – 1.48	0.33
**Served in an area of conflict**	1.05	0.67 – 1.64	0.83

**Served in Vietnam**	2.12	0.88 – 5.06	0.09
**Served in Korea**	0.48	0.14 – 1.63	0.24
**Served in SE Asia**	1.01	0.62 – 1.66	0.96
